# The association between BMI and body weight perception among children and adolescents in Jilin City, China

**DOI:** 10.1371/journal.pone.0194237

**Published:** 2018-03-26

**Authors:** Yun Wang, Hongjian Liu, Fangyuan Wu, Xiaodi Yang, Mengjia Yue, Yingxin Pang, Xuanxuan Li, Juan Ma, Ge Zhou, Ping Gong, Meitian Liu, Xiumin Zhang

**Affiliations:** 1 Department of Epidemiology and Biostatistics, School of Public Health, Jilin University, Changchun, Jilin, China; 2 Department of Social Medicine and Health Management, School of Public Health, Jilin University, Changchun, Jilin, China; University of Kansas Medical Center, UNITED STATES

## Abstract

**Objectives:**

We evaluated the association between BMI and body weight perception in a sample of children and adolescents.

**Methods:**

A cross-sectional school-based study was conducted among 7–18 year-olds (N = 9727) from 4 districts in Jilin City, China. We calculated BMI from measured weight and height and assessed body weight perception using a single questionnaire item. We analyzed these data using SPSS version 20.0.

**Results:**

Approximately 19.8% of these youth perceived themselves as underweight, 57.8% as normal weight, and 22.4% as overweight. In reality, 4.9% were underweight, 64.3% were normal weight, and 30.8% were overweight. Furthermore, approximately 66.4% of these Chinese youth correctly perceived their body image, 28.2% underestimated their true body image, and 5.4% overestimated their weight status. Girls were more likely than boys to overestimate their weight (*χ*^*2*^ = 135.4, p < 0.05). Adolescents 13–18 years old were more likely than children 7–12 years old to overestimate their weight (*χ*^*2*^ = 248.4, p < 0.05). Senior high school students were the most likely to overestimate their weight (*χ*^*2*^ = 297.6, p < 0.05). Kappa tests revealed significant differences in consistency analysis of BMI and body weight perception (*Kappa* = 0.352, p < 0.05). *Kappa* < 0.4, the consistency of BMI and body weight perception was poor.

**Conclusions:**

A mismatch existed between BMI and body weight perception among these children and adolescents. Thus, schools and parents should take steps to help them improve weight management and overall health awareness.

## Introduction

Adolescence is a crucial time in the profound development of physical and psychological health [1]. During the growth spurt, as significant changes occur the body, weight perception is also influenced. Body weight perception refers to one's estimate of body image with all of the accompanying feelings, attitudes, and thoughts concerning weight, size, shape, and appearance. Body weight perception plays a significant role in weight management [2–5]. A previous study has concluded that body weight perception is a better predictor of body management and related behaviors than body mass index (BMI), ie, actual weight status [6]. However, in both Eastern and Western countries, some related studies have shown that the correlation of body weight perception and BMI is relatively poor [3,7,8]; many children and adolescents are unable to perceive their weight status accurately. Almost one-third of adolescents misperceive their body weight; moreover, compared to boys, girls are more likely to hold misperceptions (27.3% vs.42.2%) [9]. Children and adolescents promote some weight control practices [10–11] and change eating habits [4] on the basis of body weight perception. For example, it is appropriate to gain weight when persons are actually underweight or lose weight when actually overweight. Persons who have weight misperception are more likely than their accurate recognition counterparts to develop some physical (hypertension, hyperlipidemia, diabetes mellitus type 2, metabolic syndrome) [12–13] and psychological (distress, depression, self-abasement, anxiety, agrypnia) [14–15] problems. In particular, adverse psychological consequences seem to be more strongly associated with body weight perception, regardless of BMI [16–17].

In China, policy reforms in the last couple of decades have brought great changes in people’s lifestyles. These dramatic shifts have accelerated an increase in the prevalence of overweight or obesity [18]. Exposed to the variables of dietary patterns, leisure activities, and the "clash" of Eastern and Western cultures, Chinese children and adolescents may be prone to have difficulty estimating their true weight status and express dissatisfaction concerning their body weight and shape [19]. Some studies report that perception of body image may be related to mass media and evolving cultural beliefs about beauty, such as is found through television programs, cartoons, and magazines [20–22]. However, studies on the discrepancy between body weight perception and actual weight in Chinese children and adolescents are relatively limited. Therefore, it is necessary to assess the association between BMI and body weight perception. The assessment included perceived weight category, estimated BMI category based on measured weight and height, and the association between these estimates. We also investigated how BMI, body weight perception, and their association varies by age, sex, residence (urban/rural), and school status (ie, primary school, junior high school, senior high school). Previous studies have concluded that girls are more likely than boys to perceive themselves as overweight even though they are actually of normal weight, or even underweight [2,19]. Urban youth tend to weigh more and more readily assess themselves as having an abnormal body image compared to rural youth [4]. Because school status differences have seldom been studied with Chinese youth, in our study we examined differences from primary school to senior high school by sex.

## Methods

### Participants

A cross-sectional school-based study was conducted among 9727 children and adolescents (7–18 years) from urban and rural area of 4 districts in Jilin City, China between April 2015 and June 2015. A stratified cluster sample was used. We randomly selected 24 schools from 4 districts (2 urban and 2 rural) according to socioeconomic status, school size, and the quality of education. Six schools were selected from each district (primary school from grade 1 to grade 6, junior high school from grade 7 to grade 9, senior high school from grade 10 to grade 12). In principle, two classes were selected randomly in each grade of each school. However, the actual sampling should be adjusted according to the actual situation, in which if the number of classes in a school is not enough, we will add one more school. In fact, all except one rural school had at least two classes in every grade, and we randomly added another school in the corresponding area.

### Ethical standards

This study including methods of informed consent was reviewed and approved by the Ethics Committee of Jilin University School of Public Health (Reference Number: 2014-04-01), Written informed consent was obtained from all participants and their parents or legal guardians before their participation in the survey.

### Instruments and study variables

This study included a face-to-face questionnaire (sociodemographic information) and physical measurement (height and weight). Sociodemographic information included sex (girls and boys), age (years), residence (urban and rural) and school category (from primary school to senior high school). Height and weight were measured by trained investigators using standardized protocols. These processes were completed in their classrooms during class time; if students had problems interpreting the items, one of the investigators provided assistance. In addition, all participants finished these processes except those students who were absent from school.

#### BMI (body mass index)

BMI was calculated by weight divided by height squared (kg/m^2^). We calculated overweight according to the International Obesity Task Force (IOTF) age-and sex-adjusted cutoff values [23]. Because IOTF does not provided BMI cutoff values for underweight, our underweight estimates were based on World Health Organization Growth Reference 2007 documentation [24].

#### Body weight perception

Body weight perception was measured using a single questionnaire item: What do you think of your body size? with the following 3 response options: underweight, normal weight, overweight. We designed this item for the purpose of the survey.

#### Association of BMI and body weight perception

The association of BMI and body weight perception was subdivided into 3 categories: 1/Underestimation, 2/Consistency (defined as being either underweight- perceiving underweight, normal weight- perceiving normal weight, or overweight- perceiving overweight), 3/Overestimation.

### Data analysis

Statistical analyses were carried out using SPSS (version 20.0). Accounting for sampling stratification, primary sampling units, and clustering, we weighted all proportions to obtain results representative of Jilin City. First, descriptive analyses (percentage, sum, mean and standard deviation) were used to describe the sample. Second, separate analyses were conducted for factors presumed to be of importance (sex, age, residence, and school category) to address differences in frequency distribution of BMI and body weight perception. Moreover, Rao–Scott *χ*^*2*^ tests were conducted to compare differences between the consistency of BMI and body weight perception both adjusted for sex, age, residence, and school category. All associations with p values < 0.05 were considered to be statistically significant.

## Results

Table 1 provides the descriptive characteristics of the sample. We investigated Chinese children and adolescents, 7–18 years old from urban (53.2%) and rural (46.8%) areas of 4 districts in Jilin City; the sample included 49.8% boys and 50.2% girls. The mean age was 12.7 years (12.5 and 12.9 years for boys and girls respectively). We considered 2 age groups: 7–12 years (45.8%) and 13–18 years (54.2%). The mean BMI was 20.5 kg/m^2^ for the overall sample (20.9 for boys and 20.1 kg/m^2^ for girls). School category was subdivided into primary school (39.4%), junior high school (30.6%), and senior high school (30.0%). For actual weight groups, BMI calculated by measured weight and height revealed 4.9% to be underweight, 30.8% to be overweight; however, 19.8% perceived themselves as underweight and 22.4% perceived themselves as overweight.

**Table 1:** Descriptive characteristics of the sample.

	Boys (N = 4843)	Girls (N = 4884)	Total (N = 9727)
Age (years, Mean, SD)	12.5(3.2)	12.9(3.3)	12.7(3.3)
Weight (kg, Mean, SD)	54.5(20.2)	49.2(15.4)	51.8(18.2)
Height (cm, Mean, SD)	158.7 (17.7)	154.7(13.5)	156.7(15.9)
BMI (kg/m^2^, Mean, SD)	20.9(4.8)	20.1(4.2)	20.5(4.5)
Age (years, N, %)			
7–12	2344(48.4)	2107(43.1)	4451(45.8)
13–18	2499(51.6)	2777(56.9)	5276(54.2)
School category (N, %)			
Primary school	2039(42.1)	1793(36.7)	3832(39.4)
Junior high school	1541(31.8)	1438(29.4)	2979(30.6)
Senior high school	1263(26.1)	1653(33.8)	2916(30.0)
Residence (N, %)			
Urban	2532(52.3)	2639(54.0)	5171(53.2)
Rural	2311(47.7)	2245(46.0)	4556(46.8)
BMI category (N, %)			
Underweight	239(4.9)	240(4.9)	479(4.9)
Normal weight	2779(57.4)	3475(71.2)	6254(64.3)
Overweight	1825(37.7)	1169(23.9)	2994(30.8)
Weight perception category (N, %)			
Underweight	1089(22.5)	834(17.1)	1923(19.8)
Normal weight	2630(54.3)	2995(61.3)	5625(57.8)
Overweight	1124(23.2)	1055(21.6)	2179(22.4)

Table 2 provides the descriptive characteristics of the sample in body weight perception and BMI category. In the group of body weight perception, A little more than half of participants (57.8%) evaluated themselves as being of normal weight, 19.8% evaluated themselves as being underweight, 22.4% evaluated themselves as being overweight. However, in the group of BMI, 64.3% were normal weight, 4.9% were underweight, and 30.8% were overweight. Rao–Scott *χ*^*2*^ tests showed significant differences in body weight perception by age (*χ*^*2*^ = 88.2, p < 0.05), sex (*χ*^*2*^ = 45.1, p < 0.05), and school category (*χ*^*2*^ = 122.3, p < 0.05). Compared with girls and 7–12 years group, boys and 13–18 years group were more likely to perceive themselves as overweight. Rao–Scott *χ*^*2*^ revealed significant differences in BMI category by age (*χ*^*2*^ = 44.3, p < 0.05) and sex (*χ*^*2*^ = 210.6, p < 0.05). For BMI category, compared with girls and 13–18 years group, boys and 7–12 years group were actually more overweight. For both body weight perception and BMI category, there was no significant difference between urban and rural areas.

**Table 2:** Demographic characteristics of the sample in body weight perception and BMI category^a^.

	Total (N,%)	Body weight perception (N,%)			*χ*^*2*^	*p*	BMI (N,%)			*χ*^*2*^	*p*
		Underweight	Normal weight	Overweight			Underweight	Normal weight	Overweight		
**All respondents**		1923(19.8)	5625(57.8)	2179(22.4)			479(4.9)	6254(64.3)	2994(30.8)		
**Sex**											
Boys	4843(49.8)	1119(23.1)	2641(54.5)	1083(22.4)	45.1	**0.049**	195(4.0)	2765(57.1)	1883(38.9)	210.6	**0.007**
Girls	4884(50.2)	1002(20.5)	2987(61.1)	895(18.3)			205(4.2)	3448(70.6)	1231(25.2)		
**Age (years)**											
7–12	4451(45.8)	1035(23.3)	2603(58.5)	813(18.3)	88.2	**0.022**	149(3.4)	2825(63.5)	1477(33.2)	44.3	**0.048**
13–18	5276(54.2)	950(18.0)	2932(55.6)	1394(26.4)			329(6.2)	3365(63.8)	1582(30.0)		
**Residence**											
Urban	5171(53.2)	944(18.3)	3158(61.1)	1069(20.7)	43.3	0.064	184(3.6)	3108(60.1)	1879(36.3)	41.6	0.098
Rural	4556(46.8)	1088(23.9)	2543(55.8)	925(20.3)			202(4.4)	2984(65.5)	1370(30.1)		
**School category**											
Primary school	3832(39.4)	919(24.0)	2230(58.2)	683(17.8)	122.3	**0.027**	132(3.5)	2445(63.8)	1255(32.7)	43.0	0.088
Junior high school	2979(30.6)	491(16.5)	1719(57.7)	769(25.8)			160(5.4)	1826(61.3)	993(33.3)		
Senior high school	2916(30.0)	560(19.2)	1546(53.0)	810(27.8)			190(6.5)	1994(68.4)	732(25.1)		

^a^ Weighted for complex survey was used in the statistical analysis.

To assess the association of BMI and body weight perception, both permutations were divided into 3 groups: underestimation, consistency, and overestimation (Table 3). Among participants, 28.2% underestimated their weight status, 66.4% accurately perceived their weight status, and 5.4% overestimated their weight status. Rao–Scott *χ*^*2*^ tests revealed significant differences in consistency of BMI and perceived weight category by sex (*χ*^*2*^ = 135.4, p < 0.05), age (*χ*^*2*^ = 248.4, p < 0.05), and school category (*χ*^*2*^ = 297.6, p < 0.05). Boys were less likely than girls to overestimate their weight. Members of the 13–18 year-old group were more likely than their 7–12 year-old counterparts to overestimate their weight, and students in senior high school was the most likely to overestimate their weight. In addition, there was no significant difference between urban and rural areas in consistency of BMI and body weight perception.

**Table 3:** Consistency of BMI and body weight perception by sex, age, residence, and school category^a^.

	Total	Underestimation		Consistency		Overestimation		*χ*^*2*^	*p*
		N (%)		N (%)		N (%)			
**All respondents**	9727	2746(28.2)		6457(66.4)		524(5.4)			
**Sex**									
Boys	4843	1822(37.6)		2901(59.9)		120(2.5)		135.4	**0.001**
Girls	4884	1346(27.6)		3300(67.6)		238(4.8)			
**Age (years)**									
7–12	4451	1644(36.9)		2688(60.4)		119(2.7)		248.4	**<0.001**
13–18	5276	1129(21.4)		3818(72.4)		329(6.2)			
**Residence**									
Urban	5171	1730(33.5)		3262(63.0)		179(3.5)		1.3	0.756
Rural	4556	1479(32.5)		2908(63.8)		169(3.7)			
**School category**									
Primary school	3832	1440(37.6)		2287(59.7)		105(2.7)		297.6	**<0.001**
Junior high school	2979	684(23.0)		2165(72.7)		130(4.4)			
Senior high school	2916	563(19.3)		2068(70.9)		285(9.8)			

^a^ Weighted for complex survey was used in the statistical analysis.

Table 4 provides consistency analysis of BMI and body weight perception. Kappa tests revealed significant differences in consistency analysis of BMI and body weight perception (*Kappa* = 0.352, p < 0.05). *Kappa* < 0.4, the consistency of BMI and body weight perception was poor. In fact, 77.8% who were actually underweight perceived themselves as underweight, 67.2% who were actually normal weight perceived themselves as normal weight, and 54.6% who were actually overweight perceived themselves as overweight.

**Table 4:** Consistency analysis of BMI and body weight perception^a^.

BMI	Total	Body weight perception			*Kappa*	*P*
		Underweight N (%)	Normal weight N (%)	Overweight N (%)		
Underweight	479	373(77.8)	98(20.5)	8(1.7)	0.352	**<0.001**
Normal weight	6254	1785(28.5)	4203(67.2)	266(4.3)		
Overweight	2994	48(1.6)	1311(43.8)	1635(54.6)		

^a^ Weighted for complex survey was used in the statistical analysis.

## Discussion

In our study, there was a significant difference between BMI and body weight perception in Chinese youth 7–18 years old. Overall, 33.6% of the participants misperceived their body image, with 28.2% underestimating their weight status, and 5.4% overestimating their weight status. These results are similar to previous studies between BMI and body weight perception among children and adolescents [19,25–26]. We found that boys are poorer than girls at estimating their body image, a finding consistent with previous studies [8,27]; however, these findings were different from some prior observations in which boys estimated their body image more correctly than girls [28–29], or demonstrated body image misperceptions to be approximately equal across the sexes [30]. On the other hand, girls tended to overestimate their weight and selected a thinner ideal body shape; in other words, boys were more likely than girls to underestimate their weight and preferred to select a strong ideal body shape, which may be influenced by peers, mass media, parents, and other sociocultural factors that promote slimness for girls [7,19] and muscularity for boys [2,31], even though such pursuits may be unreasonable or unhealthy [32]. The observation is similar to previous studies about the association of BMI and body weight perception among Chinese [33] and American [8] children and adolescents.

In our study we also found that accurate perception was better in adolescents than children. It is not surprising, as adolescents may have had more chances to be exposed to messages about body weight [8]. However, compared to children, adolescents were more likely to perceive overweight when they were actual normal weight or underweight. Similarly, the above phenomenon was also applicable to students in senior high school as opposed to primary school or junior high school. It may be related to caring about their appearance because dating begins around this stage and many students will begin to fall in love [34].

Previous studies have shown significant current-ideal body image gaps in accuracy of BMI and body weight perception between urban and rural youth [35–36]. Compared to children and adolescents in rural areas, urban youths tended to be overweight but consider themselves as being underweight or overweight, regardless of their actual weight status [19,37]. However, this study revealed no significant difference between urban and rural areas. The reason may be the following factors: (1) In recent years, with the continuous development of China's economy and urbanization, the standard of living in rural areas and the income of families have been continuously increasing. Parents in rural areas have the ability to provide children and adolescents with more and better nutrition. (2) In our study, most of the rural areas are located in the suburbs and towns with few rural villages, so the conditions in all aspects are relatively good and close to the level of urban areas.

Adolescence is not only a remarkable time in terms of physical and psychological development, but also an important period for development of healthy behaviors that track into adulthood. As an important part of body shape, body weight perception is a component of actual weight dissatisfaction and weight concerns [38]. Thus, misperception may potentially affect children and adolescents to adopt unhealthy lifestyle such as smoking, drinking, and poor eating habits to balance their weight. Therefore, we should take necessary measures to impart knowledge about health and proper weight management that contributes to accurate assessment of body image.

School is a crucial institution for the development of physical and psychological health of children and adolescents. This school-based survey focused on the association between BMI and body weight perception in a large sample of children and adolescents (from grade 1 to grade 12). The results showed that a mismatch exists between BMI and body weight perception. Therefore, schools and parents should take targeted measures to help youth improve weight management and increase their awareness of health. First, school leaders, teachers, and parents should be trained to increase awareness of health and help students strengthen their behaviors and build a favorable growth environment for students. Second, in China, because of entrance pressure, a lot of activity classes have been canceled. So, it is necessary for schools to carry out more extracurricular activities and encourage physical exercise. Moreover, students should be taught health knowledge that will contribute to improved accuracy of body image and weight management and lead to reasonable eating habits.

In our study, some limitations must be noted. First, the research was cross-sectional so causality was not able to be determined. Second, we assessed body weight perception with a single question; one item may be inadequate for describing body image. Third, we only assessed the association between BMI and body weight perception by age, sex, residence, and school category. Future studies should investigate associations among eating habits, weight dissatisfaction and concerns, physical activity, and psychological symptoms.

Despite these limitations, our survey still has many advantages. (1) This study is a large, regionally representative sample and the results provide useful information for policy makers in making informed decisions to promote the healthy growth of children and adolescents in this area. (2) There are significant regional differences in the population between this study and previous studies. Previous studies were mainly concentrated in the southern cities of China, This study was conducted in Jilin City, which is a city of northeastern China. Furthermore, there are few large sample surveys on overweight and obesity among children and adolescents in the northeast of China. (3) School status differences have seldom been studied with Chinese youth, in our study we examined differences from primary school to senior high school by sex. (4) We estimated BMI (actual weight) by measuring precise height and weight, rather than relying on self-reported height and weight. (5) We analyzed the association between BMI and body weight perception and emphasized the importance of body weight perception. Misperception is harmful to the health and normal growth of young people. Thus, sufficient social efforts are crucial for improving physical qualities and physical and mental health for children and adolescents.

## Conclusions

Our study showed that a discrepancy exists between BMI and body weight perception. Girls, youth 13–18 years of age, and senior high school youth were more likely than their respective counterparts to overestimate their weight. Therefore, schools and parents should take targeted measures to help children and adolescents improve their weight management and increase their awareness of actual weight categories, thereby contributing to improved accuracy of body image, and in turn, reasonable eating habits and physical activities.

## Supporting information

S1 File Survey questionnaire in Chinese. (DOC)

S2 File Survey questionnaire in English. (DOC)

S1 Database The database for this study. (SAV)
