# Supplementary material for: The association between BMI and body weight perception among children and adolescents in Jilin City, China
Source: PLoS One. 2018 Mar 26;13(3):e0194237. doi: 10.1371/journal.pone.0194237 (PMC5868793; doi:10.1371/journal.pone.0194237)
Supplement: S1 File — (DOC) [file pone.0194237.s001.doc]

**编号：□ □ □ □ □□ □□**

区/县 学校类型 学校编码 年级 班级 学生编号

本页检测记录由工作人员填写

**检测结果记录**

1. 身体测量 （保留小数点后1位）

（1a）身高： cm（不穿鞋、不戴帽）

（1b）体重： kg（不穿外衣和鞋）

2. 视力测量

（2a）裸眼左眼视力(3米远视力检查)：

（2b）裸眼右眼视力(3米远视力检查)：

**调查员**（签名）：

**调查日期**： 年 月 日

**学生问卷**

请**同学们**回答下列问题，并在与自己实际情况相符合的选项上直接打“√”。

1姓名： 年级 班

2性别： ① 男 ② 女 3出生日期：　　 　年　　 　月　　　 日

4你认为你目前的体重是： ①偏瘦 ②适中 ③偏胖

5你认为你目前的视力情况是： ①正常 ②视力不良（如：近视、远视、散光等）

6你是否经常连续看电视、电脑或玩手机超过40分钟？ ①是 ②否

7连续看电视，电脑或玩手机超过40分钟后，你会停下休息眼睛吗？ ①是 ②否

8你有过在晚上关灯的情况下，玩手机或看电视、电脑吗？ ①是 ②否

9你有过长时间用眼后，感觉眼睛不舒服仍继续用眼的情况吗？ ①是 ②否

10你在看书时总能保持眼与书之间30厘米以上的距离吗？ ①是 ②否

11你平时是否有躺着看书，乘车或走路看书的习惯？ ①是 ②否

12你平时看书或写作业时的坐姿，能做

到**下图**中那样并保持吗？

①是 ②否


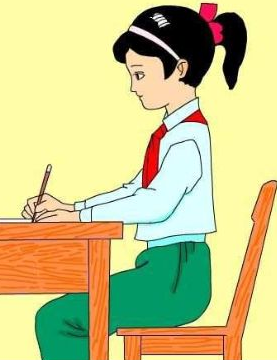


13你平时写字的握笔姿势能做到**下图**中那样并保持吗？

①是 ②否


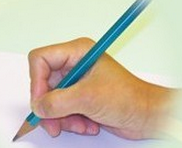


14老师或家长是否经常提醒你注意读书写字姿势？ ①是 ②否

15你是否定期调换座位？ ①是 ②否

16你在读书写字时，觉得教室里的桌椅高度如何？ ①偏高 ②适中 ③偏低

17你在读书写字时，觉得教室里的灯光亮度如何？ ①偏亮 ②适中 ③偏暗

18你戴眼镜的情况是？ ①不戴眼镜 ②看不清时戴 ③全天配戴
