# Supplementary material for: The association between BMI and body weight perception among children and adolescents in Jilin City, China
Source: PLoS One. 2018 Mar 26;13(3):e0194237. doi: 10.1371/journal.pone.0194237 (PMC5868793; doi:10.1371/journal.pone.0194237)
Supplement: S2 File — (DOC) [file pone.0194237.s002.doc]

**Code： □ □ □ □ □□ □□**

District / County School Category School Code Grade Class Student Code

Results will be filled by staff

Examination results

1.Physical measurement (Retain 1 digit after the decimal point)

（1a）Height： cm (No shoes, no hats)

（1b）Weight： kg (No coats, no shoes)

2. Visual acuity measurement

（2a）Uncorrected left eye vision (Visual acuity examination at 3 meters):

（2b）Uncorrected right eye vision (Visual acuity examination at 3 meters):

Investigator (signature）：

Survey date(y/m/d)：

**Student questionnaire**

Please answer the following questions, and in accord with their actual options directly on tick“√”.

1 Name： Grade Class

2 Sex： ①Boy ②Girl 3 Date of birth(y/m/d)：

4 What do you think of your body size? ① Underweight ② Normal weight ③ Overweight

5 What do you think of your current vision?：

①Normal ②Poor sight (Such as: myopia, hyperopia, astigmatism, etc.)

6 Do you often watch TV, constantly use computer or mobile phone for more than 40 minutes?

①Yes ②No

7 After watching TV, constantly using computer or mobile phone for more than 40 minutes, do you stop to rest your eyes? ①Yes ②No

8 Have you ever watched TV, used computer or mobile phone with lights turned out?

①Yes ②No

9 Have you ever felt uncomfortable with your eyes after long hours of study but still don’t stop to rest?

①Yes ②No

10 Do you always maintain a distance of 30 cm or more between the eye and the book when reading?

①Yes ②No

11 Do you have the habit of reading while lying in the bed, being on a vehicle or walking?

①Yes ②No

12 When you read or write your homework, can you do it as it is in the following picture and keep it?

①Yes ②No


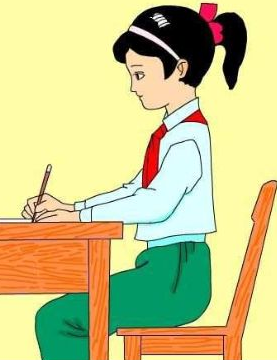


13 Do you usually hold the pen's position as shown in the following picture and keep it?

①Yes ②No


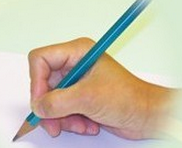


14 Do teachers or parents often remind you to pay attention to reading and writing posture? ①Yes ②No

15 Do you change seats regularly? ①Yes ②No

16 What do you think of the height of desks and chairs in the classroom when reading or writing?

① High ② Moderate ③ Low

17 How bright is the light in the classroom when you are reading or writing?

①Light ②moderate ③dark

18 When do you wear glasses?

①Do not wear glasses ②Wear glasses when you can't see clearly ③Wear glasses all day
